# Long-term complete remission with Ipilimumab in metastatic castrate-resistant prostate cancer: case report of two patients

**DOI:** 10.1186/s40425-017-0232-7

**Published:** 2017-04-18

**Authors:** Luc Cabel, Elika Loir, Gwenaelle Gravis, Pernelle Lavaud, Christophe Massard, Laurence Albiges, Giulia Baciarello, Yohann Loriot, Karim Fizazi

**Affiliations:** 1 Institut Gustave Roussy, University of Paris-Sud, Department of Cancer Medicine, 114 Rue Edouard Vaillant, 94800 Villejuif, France; 2 Institut Paoli-Calmettes, Department of Medical Oncology, 13009 Marseille, France

**Keywords:** Ipilimumab, Metastatic castrate-resistant prostate cancer, Immunotherapy

## Abstract

**Background:**

Prostate cancer is one of the most common cancers in men and the fourth leading cause of cancer mortality worldwide. Although major progress has been achieved in the last years for patients with metastatic castrate-resistant prostate cancer (mCRPC), thanks to next-generation androgen receptor axis targeted drugs, taxanes, and bone-targeted agents, immunotherapy has not been widely approved and used for the treatment of prostate cancer. Two large studies with ipilimumab, an anti-CTLA-4 (cytotoxic T-lymphocyte antigen 4) antibody reported improved progression-free survival, but not statistically improved overall survival at the primary analysis (CA184 043 and CA184 095).

**Case presentation:**

Here, we report on two patients who received ipilimumab in these trials and are still in long-term complete remission with a follow-up of 64 and 52 months respectively after the initiation of ipilimumab. Immunohistochemical staining for hMLH1, hMSH2, hMSH6 and PMS2 was performed on archival prostate biopsy samples from one of the two patients; they exhibited normal protein expression. Interestingly for this patient, a high CD3+ and CD8+ T cell infiltration was observed on archival prostate biopsies as well as Treg FoxP3+ T cells.

**Conclusion:**

Ipilimumab produces clinical activity in patients with CRPC, including very long responders with no detectable residual disease.

## Background

Prostate cancer is one of the most frequent cancers in men and the fourth leading cause of cancer mortality worldwide [1]. Several treatments have yielded improved survival in metastatic castrate-resistant prostate cancer (mCRPC): cytotoxic chemotherapy (docetaxel and cabazitaxel), next-generation androgen receptor pathway targeting agents (abiraterone acetate and enzalutamide), and bone-targeted agents (radium-223). These agents are recommended by guidelines and widely used [2–4]. However, despite this expanding armamentarium yielding longer survival, mCRPC remains an incurable disease.

The use of the only immunotherapy with associated improved survival, Sipuleucel T, an autologous cellular immunological agent, is currently restricted to the US [5, 6]. One of the most interesting effects of immunotherapy is the potentially long duration of remission in responders, observed in melanoma [7], lung cancer [8] and renal cell carcinoma [9], with some patients still in complete remission years later. Ipilimumab is a humanized IgG1 monoclonal antibody that binds to the cytotoxic T-lymphocyte antigen-4 (CTLA-4) regulatory receptor on T cells. As such, it is an immune checkpoint inhibitor promoting the maturation of CD8+ cell effectors and depleting regulatory T cells. It is currently approved for the treatment of patients with melanoma after an enhancement of overall survival was achieved when it was administered alone [7] or in combination with nivolumab [10] (an anti-PD1 antibody). Two phase III trials testing ipilimumab have been conducted in men with mCRPC [11, 12]. The first reported, CA184 043, accrued patients who had previously received docetaxel [11], while the second trial, CA184 095, enrolled chemotherapy-naive and asymptomatic or minimally symptomatic patients with mCRPC [12]. In CA184 043, 799 patients were randomized 1:1 to receive bone-directed radiotherapy (8 Gy in one fraction) followed by either ipilimumab 10 mg/kg or a placebo every 3 weeks for up to four injections. Non-progressors could continue to receive ipilimumab at 10 mg/kg or a placebo as maintenance therapy every 3 months until disease progression, an unacceptable toxic effect, or death. The primary analysis of this trial reported non-significantly improved overall survival (hazard ratio [HR] 0 · 85, 0 · 72-1 · 00; *p* = 0 · 053). However, evidence of efficacy was provided with improved progression-free survival (hazard ratio 0.70, 0.61–0.82; *p* < 0.0001) in the ipilimumab arm [11], and also improved PSA response rate in the ipilimumab arm (13.1%, 9.5–17.5 versus 5.2%, 3.0–8.4). In a second post-hoc analysis performed with an additional year of follow-up, the overall survival trend favouring the ipilimumab + radiotherapy arm was maintained (HR = 0.84 (0.72–0.98), *p* = 0.03, for overall survival) [13]. Data from the final, long-term analysis are expected soon.

The CA184 095 study tested single-agent ipilimumab (without radiotherapy) in mCRPC patients with less advanced disease. A total of 602 asymptomatic or minimally symptomatic patients with chemotherapy-naive mCRPC and no known visceral metastases were randomized: 400 patients in the ipilimumab arm (10 mg/kg every 3 weeks for up to four injections, then 10 mg/kg every 3 months in non-progressors) and 202 patients in the placebo arm. Again, overall survival was not different (hazard ratio, 1.11; 95.87% CI, 0.88 to 1.39; *P* = .3667) in this trial, although progression-free survival and PSA response rate were improved in the ipilimumab arm (progression-free survival: hazard ratio, 0.67; 95.87% CI, 0.55 to 0.81; PSA response rate with ipilimumab (23%; 95% CI, 19–27%) versus placebo (8%, 95% CI, 5–13%). Also, patients in the ipilimumab arm achieved a higher prostate-specific antigen (PSA) response rate (23%), than those in the placebo arm (8%). Nine (2%) deaths occurred in the ipilimumab arm due to treatment-related adverse events (AEs) and immune-related grade 3 to 4 AEs occurred in 31 and 2% of the patients, respectively.

Here, we present two patients who were enrolled in the ipilimumab arm of the above-mentioned trials and who are still in long-term complete remission.

## Case presentation

### Case 1

A 51-year old man was diagnosed in August 2009 with a Gleason 8 prostate cancer with multiple synchronous bone metastases. His serum PSA level was 225 ng/mL. An LH-RH agonist (goserelin) was started, and his PSA declined to 3.5 ng/mL. In August 2010, after 10 months on androgen deprivation therapy (ADT), resistance to castration developed and he received chemotherapy with docetaxel and prednisone given 3 weekly, which resulted in a PSA decline (from 135 to 90 ng/mL). Docetaxel was discontinued after 8 cycles due to nail toxicity in May 2011. In June 2011, he experienced cancer progression with a PSA rise, progression of bone and lymph node metastases on imaging, and bone pain requiring opioids. He was then enrolled on the CA184 043 trial [11] and was randomly assigned to the ipilimumab arm. His only co-medication was oxycodone for bone pain.

The patient received single-fraction radiotherapy (8 Gy delivered to vertebrae, T8 to T11) the day before starting intravenous ipilimumab (10 mg/kg every 3 weeks). The baseline PSA level was 118 ng/mL, serum alkaline phosphatase level was 2.02-fold the upper limit of normal, and haemoglobin was 129 g/L.

One week after treatment initiation, he presented with urinary incontinence, ataxia and decreased lower limb sensitivity. The diagnosis of spinal cord compression at T9 level was made. He was treated with a high-dose corticosteroid infusion and underwent emergency surgery, with a favourable outcome.

He subsequently continued ipilimumab therapy, with a decrease in corticosteroid doses. After the third injection, he presented immune-related adverse events (irAE) with a grade 2 rash and grade 1 diarrhoea which again required an increase in the prednisone dose (1 mg/kg) and the episode resolved clinically. Due to the favourable evolution of the rash and diarrhea with prednisone and time, no skin or colon biopsies were realized. After the third injection, he also developed an ipilimumab infusion reaction which required premedication with high-dose corticosteroids and anti-histaminics. Prednisone was maintained at 20 mg daily for 5 months due to the initial spinal cord compression, then 10 mg daily and it was then switched to hydrocortisone.

**Fig. 1:**
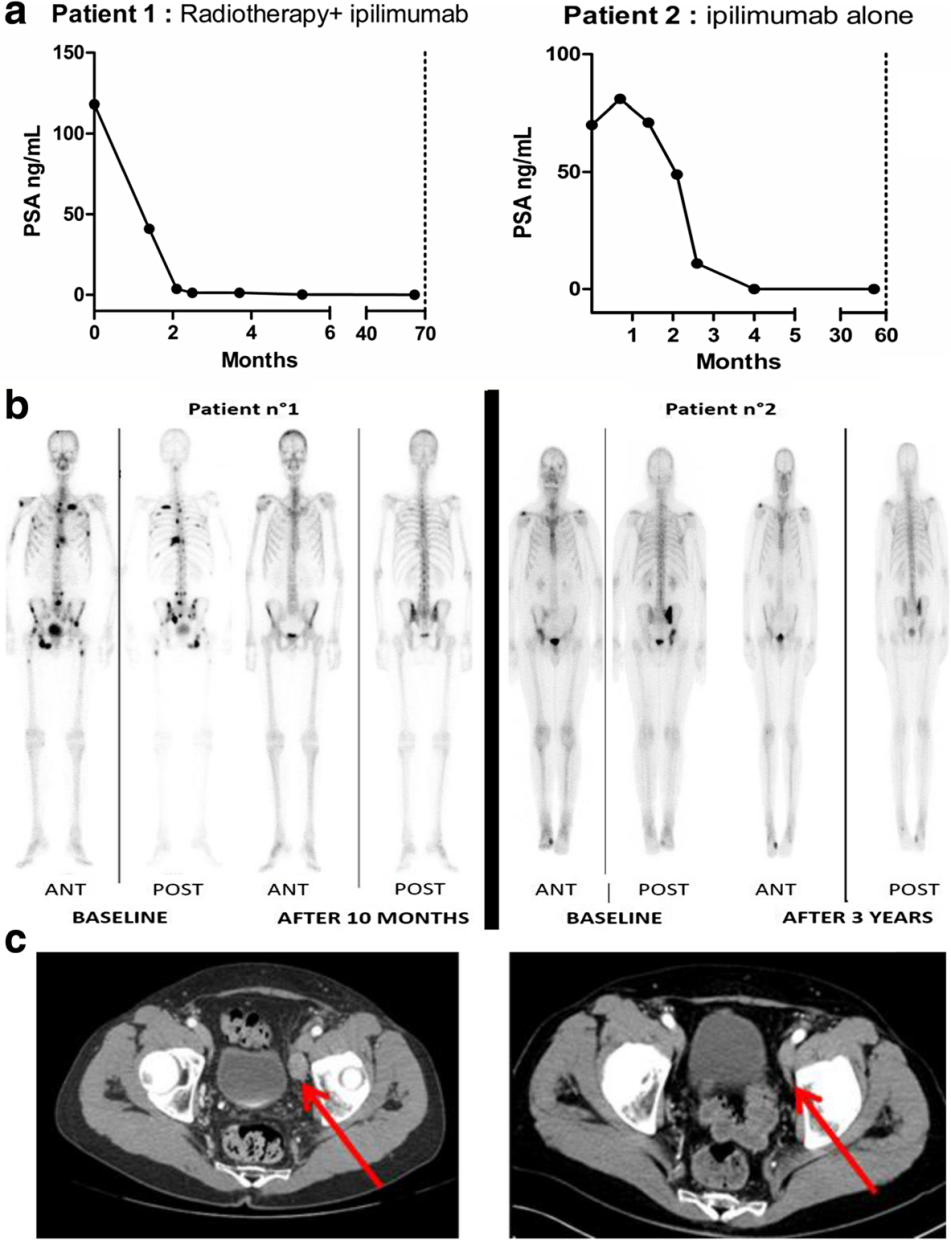
**a** PSA course after ipilimumab and radiotherapy for patient n°1 and n°2. **b** Bone scan at baseline and after treatment with ipilimumab and radiotherapy for patient n°1 and n°2. ANT = anterior, POST = posterior. **c** CT-scan for patient n°1 at baseline (*left*) and after 10 months (*right*)

After 5 months on ipilimumab, the patient permanently stopped opioids. His serum PSA level rapidly and dramatically declined at 6 weeks and remained undetectable (below 0.05 ng/mL) during follow-up (Fig. 1a). His first bone scan performed after 3 months on treatment showed decreased tracer uptake in bone metastases and no remaining pathologic uptake was detectable on subsequent bone scans, nor on the last bone scan performed 48 months after the initiation of ipilimumab. The bone scan performed after 10 months of therapy and the CT-scan are shown in Fig. 1. After almost 3 years, ipilimumab was stopped after a last injection on September 29, 2014. The patient is still asymptomatic, with a complete biochemical and morphologic response at 64 months.

**Fig. 2:**
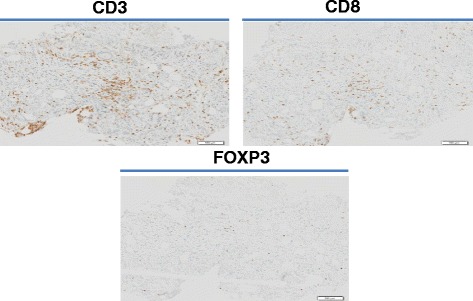
Immune infiltration in archival prostate biopsies (CD3, CD8 and FOXP3 expression by immunohistochemistry)

Immunohistochemical staining for hMLH1 (antibody from Ventana, clone M1), hMSH2 (Calbiochem, clone FE11), hMSH6 (Pharmingen, clone 44) and PMS2 (Ventana, clone EPR3947) was performed on archival prostate biopsy samples, performed at diagnosis in 2009, and showed normal protein expression. Likewise, a high CD3+ and CD8+ T cell infiltration was observed on archival prostate biopsies as well as Treg FoxP3+ T cells (Fig. 2). FoxP3 staining was highly specific with nuclear staining found only in lymphocytes and not in tumor cells.

### Case 2

In April 1996, a 49-year old man underwent a prostatectomy and a lymph node dissection for a Gleason 8, pT3a prostate cancer, with 1 positive lymph node among the 17 dissected. Adjuvant brachytherapy (8 Gy) and pelvic radiotherapy (45 Gy) were delivered. The baseline serum PSA level was 44.8 ng/mL before the prostatectomy. He experienced a PSA relapse in 1998 for which he received intermittent ADT for 7 years. In 2005, bone metastases were diagnosed, and he then received ADT continuously with the disappearance of disease on the bone scan and a marked PSA decline which ultimately became undetectable. His PSA rose to 0.85 ng/mL in December 2009 with disease stabilization when androgen receptor inhibitors (bicalutamide, nilatumide and flutamide) were sequentially added to ADT. At this time, his co-medications were only valsartan and amlodipine.

In April 2012, a further PSA rise occurred and new bone lesions were diagnosed while the patient remained asymptomatic. He was enrolled in the CA184 095 trial and received 4 injections of ipilimumab 10 mg/kg every 3 weeks from April to July 2012, followed by 1 injection every 3 months as maintenance therapy. His baseline PSA level was 70 ng/mL.

After the fourth injection, the patient experienced flare of psoriasis which required antihistaminic treatment.

The PSA level initially rose from 70 ng/mL to 81 ng/nL at 3 weeks and then declined regularly. At 13 weeks, the serum PSA concentration was undetectable (<0.01 ng/mL). Serious bone scans showed stable bone lesions. The last ipilimumab injection was given in April 2015 after 3 years of treatment. Serum PSA is still undetectable at 52 months, without any clinical or radiological sign of cancer progression (Fig. 1).

No archival tissue was available to perform a DNA mismatch repair deficiency analysis.

## Discussion

Ipilimumab produces clinical activity in patients with CRPC, including very long responders with no detectable residual disease. Long term remissions in the two major phase III clinical trials were rare, with 32 months progression-free survival rates of less than 5 and 10% respectively with ipilimumab [11, 12]. The two patients reported here did not report grade 3–4 toxicity, unlike a previous patient who also experienced a long-term sustained complete response [14], indicating that major toxicity is not required for patients to derive a major benefit from ipilimumab therapy. The most frequent irAEs in the two phase III trials (above 10% and whatever the grade) were diarrhea (39–43%), rash (17–33%), fatigue (24%), pruritus (20–27%), nausea (19%), decreased appetite (16%) and vomiting (11%). The first patient reported here experienced diarrhea, rash, and infusion reaction, though these immune-related adverse events were manageable with steroids and disappeared with time, while the second patient only experienced psoriasis, which was efficiently treated by antihistaminics. Biomarkers predicting ipilimumab efficacy are urgently needed for patients with CRPC but unfortunately only modest research has been reported so far on this subject. In melanoma, baseline high lactate dehydrogenase level, [15, 16], high neutrophils, and high neutrophil to lymphocyte ratio [17, 18] are associated with worse outcome in patients on ipilimumab, although they are not necessarily predictive of treatment efficacy. In men with CRPC receiving ipilimumab, the number of blood PD-1 expressing CD4 T lymphocytes is associated with outcome [18, 19], although it is unknown whether this parameter is predictive of a treatment benefit. Interestingly, the patient 1 had an immune infiltration of tumor by lymphocytes CD3+, CD8+ and Treg FoxP3+ T cells, which is generally associated with a better response to anti PD-1 inhibitors especially for CD8 [20]. In the epithelial compartment of tumors from 535 prostate cancer patients, 50, 44 and 58% had high immune-infiltration for CD3, CD4 and CD8, respectively [21]. A high density of CD8+ lymphocytes, especially in tumor epithelial areas, was an independent negative prognostic factor for biochemical failure-free survival. In melanoma cancer, FoxP3+ T cells infiltration was detected in 75% of evaluable pretreatment biopsies in ipilimumab-responder patients versus 36.0% in non-responders (*p* = 0.014) [22]. Tumor-infiltrating lymphocytes (TIL) in pre-treatment samples was not clearly demonstrated as a predictive biomarker in this study [22], while increases in TIL density in tumor biopsy samples collected after the second dose of ipilimumab were associated with significantly greater clinical activity [22, 23]. Recently in melanoma, CD8 + T cells infiltration and PD-L1 expression were suggested to be higher in durable ipilimumab-responders [24]. Percentages of tumor area stained for CD8 in durable responders versus non-responders were 4.3 and 1.8% respectively, whilst overall percentage of PD-L1 positive area was 11.1 and 3.7% for durable responders versus non-responders, respectively. However, none of these differences reached a statistically significant difference. In prostate cancer, two patients who had a tumor response to anti-PD1 therapy in a phase II study, had a higher infiltration of CD3 + and CD8+ T cells with higher PD-L1 expression on baseline biopsy [25]. DNA mismatch repair deficiency [26], which is associated with a high mutational load resulting in high tumor antigenicity, is a common characteristic for several immunotherapy-sensitive cancer subtypes [27, 28]. Staining was negative in one of our patients and it could not be performed due to the unavailability of cancer tissue in the second. Of note, CRPC is not believed to harbour a high mutational load [29]. Very recently, the first evidence was provided that PD1 blockade also produces anticancer activity in men with CRPC [25, 30], although it is currently unknown whether long-term complete remissions such as those reported here and elsewhere with CTLA4 blockade can be achieved.

## Conclusion

In conclusion, Ipilimumab has clinical activity in prostate cancer, as showed with the progression-free survival and PSA response rate improvement in the two large phase III studies, and can be associated with exceptional clinical benefit in rare patients. More insights in potential biomarkers predicting for benefit are urgently needed to help design the next generation of trials.

## Abbreviations

HR: Hazard ratio
mCRPC: Metastatic castrate-resistant prostate cancer
OS: Overall survival
PFS: Progression-free survival
PSA: Prostate-specific-antigen
TIL: Tumor-infiltrating lymphocytes

## References

[1] Torre LA, Bray F, Siegel RL, Ferlay J, Lortet-Tieulent J, Jemal A (2015). Global cancer statistics, 2012. CA Cancer J Clin.

[2] Horwich A, Hugosson J, de Reijke T, Wiegel T, Fizazi K, Kataja V (2013). Prostate cancer: ESMO Consensus Conference Guidelines 2012. Ann Oncol Off J Eur Soc Med Oncol.

[3] Fitzpatrick JM, Bellmunt J, Fizazi K, Heidenreich A, Sternberg CN, Tombal B (2014). Optimal management of metastatic castration-resistant prostate cancer: highlights from a European Expert Consensus Panel. Eur J Cancer Oxf Engl 1990.

[4] Gillessen S, Omlin A, Attard G, de Bono JS, Efstathiou E, Fizazi K (2015). Management of patients with advanced prostate cancer: recommendations of the St Gallen Advanced Prostate Cancer Consensus Conference (APCCC) 2015. Ann Oncol Off J Eur Soc Med Oncol.

[5] Quinn DI, Shore ND, Egawa S, Gerritsen WR, Fizazi K (2015). Immunotherapy for castration-resistant prostate cancer: progress and new paradigms. Urol Oncol.

[6] Kantoff PW, Higano CS, Shore ND, Berger ER, Small EJ, Penson DF (2010). Sipuleucel-T immunotherapy for castration-resistant prostate cancer. N Engl J Med.

[7] Robert C, Thomas L, Bondarenko I, O’Day S, Weber J, Garbe C (2011). Ipilimumab plus dacarbazine for previously untreated metastatic melanoma. N Engl J Med.

[8] Brahmer J, Reckamp KL, Baas P, Crinò L, Eberhardt WEE, Poddubskaya E (2015). Nivolumab versus Docetaxel in Advanced Squamous-Cell Non-Small-Cell Lung Cancer. N Engl J Med.

[9] Motzer RJ, Escudier B, McDermott DF, George S, Hammers HJ, Srinivas S (2015). Nivolumab versus Everolimus in Advanced Renal-Cell Carcinoma. N Engl J Med.

[10] Larkin J, Chiarion-Sileni V, Gonzalez R, Grob JJ, Cowey CL, Lao CD (2015). Combined Nivolumab and Ipilimumab or Monotherapy in Untreated Melanoma. N Engl J Med.

[11] Kwon ED, Drake CG, Scher HI, Fizazi K, Bossi A, van den Eertwegh AJM (2014). Ipilimumab versus placebo after radiotherapy in patients with metastatic castration-resistant prostate cancer that had progressed after docetaxel chemotherapy (CA184-043): a multicentre, randomised, double-blind, phase 3 trial. Lancet Oncol.

[12] Beer TM, Kwon ED, Drake CG, Fizazi K, Logothetis C, Gravis G, et al. Randomized, Double-Blind, Phase III Trial of Ipilimumab Versus Placebo in Asymptomatic or Minimally Symptomatic Patients With Metastatic Chemotherapy-Naive Castration-Resistant Prostate Cancer. J Clin Oncol. 2016;:JCO691584. 10.1200/JCO.2016.69.1584 28034081

[13] Fizazi K, Drake CG, Kwon ED, Bossi A, van den Eertwegh AJ, Scher HI (2014). Updated Overall Survival from the Phase 3 Trial, Ca184-043: Ipilimumab Vs Placebo in Patients with Post-Docetaxel Metastatic Castration-Resistant Prostate Cancer. Ann Oncol.

[14] Graff JN, Puri S, Bifulco CB, Fox BA, Beer TM (2014). Sustained complete response to CTLA-4 blockade in a patient with metastatic, castration-resistant prostate cancer. Cancer Immunol Res.

[15] Diem S, Kasenda B, Martin-Liberal J, Lee A, Chauhan D, Gore M (2015). Prognostic score for patients with advanced melanoma treated with ipilimumab. Eur J Cancer Oxf Engl 1990.

[16] Kelderman S, Heemskerk B, van Tinteren H, van den Brom RRH, Hospers GAP, van den Eertwegh AJM (2014). Lactate dehydrogenase as a selection criterion for ipilimumab treatment in metastatic melanoma. Cancer Immunol Immunother.

[17] Ferrucci PF, Ascierto PA, Pigozzo J, Del Vecchio M, Maio M, Antonini Cappellini GC (2016). Baseline neutrophils and derived neutrophil-to-lymphocyte ratio: prognostic relevance in metastatic melanoma patients receiving ipilimumab. Ann Oncol Off J Eur Soc Med Oncol.

[18] Martens A, Wistuba-Hamprecht K, Geukes Foppen MH, Yuan J, Postow MA, Wong P, et al. Baseline peripheral blood biomarkers associated with clinical outcome of advanced melanoma patients treated with ipilimumab. Clin Cancer Res Off J Am Assoc Cancer Res. 2016;22:2908–18. 10.1158/1078-0432.CCR-15-2412 PMC5770142 26787752

[19] Kwek SS, Lewis J, Zhang L, Weinberg V, Greaney SK, Harzstark AL (2015). Preexisting levels of CD4 T cells expressing PD-1 are related to overall survival in prostate cancer patients treated with ipilimumab. Cancer Immunol Res.

[20] Tumeh PC, Harview CL, Yearley JH, Shintaku IP, Taylor EJM, Robert L (2014). PD-1 blockade induces responses by inhibiting adaptive immune resistance. Nature.

[21] Ness N, Andersen S, Valkov A, Nordby Y, Donnem T, Al-Saad S (2014). Infiltration of CD8+ lymphocytes is an independent prognostic factor of biochemical failure-free survival in prostate cancer. Prostate.

[22] Hamid O, Schmidt H, Nissan A, Ridolfi L, Aamdal S, Hansson J (2011). A prospective phase II trial exploring the association between tumor microenvironment biomarkers and clinical activity of ipilimumab in advanced melanoma. J Transl Med.

[23] Gibney GT, Weiner LM, Atkins MB (2016). Predictive biomarkers for checkpoint inhibitor-based immunotherapy. Lancet Oncol.

[24] Seremet T, Koch A, Jansen Y, Schreuer M, Wilgenhof S, Del Marmol V, et al. Molecular and epigenetic features of melanomas and tumor immune microenvironment linked to durable remission to ipilimumab-based immunotherapy in metastatic patients. J Transl Med. 2016;14. doi:10.1186/s12967-016-0990-x. 10.1186/s12967-016-0990-x PMC4971660 27484791

[25] Graff JN, Alumkal JJ, Drake CG, Thomas GV, Redmond WL, Farhad M (2016). First evidence of significant clinical activity of PD-1 inhibitors in metastatic, castration resistant prostate cancer (mCRPC). Ann Oncol.

[26] Le DT, Uram JN, Wang H, Bartlett BR, Kemberling H, Eyring AD (2015). PD-1 blockade in tumors with mismatch-repair deficiency. N Engl J Med.

[27] Rizvi NA, Hellmann MD, Snyder A, Kvistborg P, Makarov V, Havel JJ (2015). Cancer immunology. Mutational landscape determines sensitivity to PD-1 blockade in non-small cell lung cancer. Science.

[28] Schumacher TN, Schreiber RD (2015). Neoantigens in cancer immunotherapy. Science.

[29] Robinson D, Van Allen EM, Wu Y-M, Schultz N, Lonigro RJ, Mosquera J-M (2015). Integrative clinical genomics of advanced prostate cancer. Cell.

[30] Hansen A, Massard C, Ott PA, Haas N, Lopez J, Ejadi S (2016). Pembrolizumab for patients with advanced prostate adenocarcinoma: preliminary results from the KEYNOTE-028 study. Ann Oncol.

